# Functional Contribution of Elevated Circulating and Hepatic Non-Classical CD14^+^CD16^+^ Monocytes to Inflammation and Human Liver Fibrosis

**DOI:** 10.1371/journal.pone.0011049

**Published:** 2010-06-10

**Authors:** Henning W. Zimmermann, Sebastian Seidler, Jacob Nattermann, Nikolaus Gassler, Claus Hellerbrand, Alma Zernecke, Jens J. W. Tischendorf, Tom Luedde, Ralf Weiskirchen, Christian Trautwein, Frank Tacke

**Affiliations:** 1 Department of Medicine III, University Hospital Aachen, Aachen, Germany; 2 Department of Medicine I, University of Bonn, Bonn, Germany; 3 Institute of Pathology, University Hospital Aachen, Aachen, Germany; 4 Department of Medicine I, University of Regensburg, Regensburg, Germany; 5 Institute for Molecular Cardiovascular Research, University Hospital Aachen, Aachen, Germany; 6 Institute of Clinical Chemistry and Pathobiochemistry, University Hospital Aachen, Aachen, Germany; Fundação Oswaldo Cruz, Brazil

## Abstract

**Background:**

Monocyte-derived macrophages critically perpetuate inflammatory responses after liver injury as a prerequisite for organ fibrosis. Experimental murine models identified an essential role for the CCR2-dependent infiltration of classical Gr1/Ly6C^+^ monocytes in hepatic fibrosis. Moreover, the monocyte-related chemokine receptors CCR1 and CCR5 were recently recognized as important fibrosis modulators in mice. In humans, monocytes consist of classical CD14^+^CD16^−^ and non-classical CD14^+^CD16^+^ cells. We aimed at investigating the relevance of monocyte subpopulations for human liver fibrosis, and hypothesized that ‘non-classical’ monocytes critically exert inflammatory as well as profibrogenic functions in patients during liver disease progression.

**Methodology/Principal Findings:**

We analyzed circulating monocyte subsets from freshly drawn blood samples of 226 patients with chronic liver disease (CLD) and 184 healthy controls by FACS analysis. Circulating monocytes were significantly expanded in CLD-patients compared to controls with a marked increase of the non-classical CD14^+^CD16^+^ subset that showed an activated phenotype in patients and correlated with proinflammatory cytokines and clinical progression. Correspondingly, CD14^+^CD16^+^ macrophages massively accumulated in fibrotic/cirrhotic livers, as evidenced by immunofluorescence and FACS. Ligands of monocyte-related chemokine receptors CCR2, CCR1 and CCR5 were expressed at higher levels in fibrotic and cirrhotic livers, while CCL3 and CCL4 were also systemically elevated in CLD-patients. Isolated monocyte/macrophage subpopulations were functionally characterized regarding cytokine/chemokine expression and interactions with primary human hepatic stellate cells (HSC) *in vitro*. CD14^+^CD16^+^ monocytes released abundant proinflammatory cytokines. Furthermore, CD14^+^CD16^+^, but not CD14^+^CD16^−^ monocytes could directly activate collagen-producing HSC.

**Conclusions/Significance:**

Our data demonstrate the expansion of CD14^+^CD16^+^ monocytes in the circulation and liver of CLD-patients upon disease progression and suggest their functional contribution to the perpetuation of intrahepatic inflammation and profibrogenic HSC activation in liver cirrhosis. The modulation of monocyte-subset recruitment into the liver via chemokines/chemokine receptors and their subsequent differentiation may represent promising approaches for therapeutic interventions in human liver fibrosis.

## Introduction

Sustained inflammation is a common characteristic of chronic liver injury in mice and men and induces the development of liver fibrosis [1], [2]. Monocytes are circulating blood leukocytes that play important roles in the pathogenesis of inflammatory disorders, because they serve as precursors for tissue macrophages and dendritic cells [3]. Over recent years, several studies have emphasized the crucial role of infiltrating monocytes for the progression of liver fibrosis in experimental mouse models [4], [5], [6], [7], [8], [9]. It has become clear that the macrophage compartment of the liver, traditionally called ‘Kupffer cells’, is constantly replenished to a significant extent by blood monocytes [4], [10] and is greatly augmented by a vast number of infiltrating monocytes upon acute or chronic liver injury [6], [11]. During fibrosis progression in mice, monocyte-derived macrophages release cytokines perpetuating chronic inflammation as well as directly activate hepatic stellate cells (HSC), resulting in their proliferation and transdifferentiation into collagen-producing myofibroblasts [5], [6], [9]. Independent studies highlighted the importance of the chemokine receptor CCR2 and its cognate ligand monocyte-chemoattractant protein 1 (MCP-1/CCL2) for monocyte recruitment during experimental hepatic fibrosis [5], [6], [7], [8]. Moreover, CCR1 and CCR5, receptors for the chemokines CCL3/MIP1α, CCL4/MIP1β and CCL5/RANTES, promote liver fibrosis in mice [11].

Human and mouse blood each contain two main monocyte subsets, which can be distinguished by high or low Gr1 (Ly6C) expression (‘Gr1^hi^ or Gr1^lo^ monocytes’) in mice [12]. We demonstrated previously that only Gr1^hi^ monocytes are massively recruited into the murine liver upon toxic injury dependent on CCR2-mediated bone marrow egress, constituting an up to 10-fold increase in CD11b^+^F4/80^+^ intrahepatic macrophages. During chronic liver damage, Gr1^hi^ monocyte-derived cells differentiate into iNOS-producing macrophages exerting proinflammatory and profibrogenic actions [6]. At present it is unclear how these findings from mouse models precisely relate to liver diseases in humans. It is well established that the number of macrophages increases during chronic liver injury and fibrogenesis [4], but detailed phenotypic characterizations of human intrahepatic monocyte-derived cells are lacking at present. Mouse Gr1^hi^ monocytes are believed to resemble the human CD14^+^CD16^−^, and Gr1^lo^ the human CD14^+^CD16^+^ subset [12]. This assumption is based on similar expression patterns of activation markers, adhesion molecules and chemokine receptors. Namely, CCR1 and CCR2 are more highly expressed on CD14^+^CD16^−^ human and Gr1^hi^ mouse monocytes, whereas CCR5 is elevated on CD14^+^CD16^+^ human and Gr1^lo^ mouse monocytes [12], [13], [14]. It is believed that CD14^+^CD16^+^ monocytes originate from CD14^+^CD16^−^ cells and represent the more mature/differentiated monocyte subset [12].

However, some discrepancies between murine and human monocyte subpopulations have not been convincingly resolved at present. For instance, Gr1^hi^ monocytes constitute about 50% of murine monocytes, while CD14^+^CD16^−^ cells represent about 90–95% of human monocytes [12]. In mice, Gr1^hi^ monocytes are often named ‘inflammatory monocytes’ due to their preferential recruitment to sites of inflammation and their proinflammatory differentiation potential, whereas in humans the CD14^+^CD16^+^ subset has long been considered to constitute ‘inflammatory monocytes’, because it is found upregulated in many inflammatory disorders and has the potential to release high amounts of proinflammatory cytokines upon stimulation *in vitro* [3], [15]. In patients with liver cirrhosis, both increased peripheral CD14^+^CD16^−^ and CD14^+^CD16^+^ monocytes have been reported from small clinical studies [16], [17].

As the interference with monocyte subset infiltration, differentiation and activation may represent interesting novel targets for future therapeutic approaches in liver fibrosis [4], we aimed at defining the functional contributions of monocyte subpopulations to liver fibrogenesis in humans. Our study, comprising 226 patients with chronic liver diseases (CLD) at various stages of fibrosis/cirrhosis from different disease etiologies and 184 controls, demonstrates that circulating monocytes increase during disease progression, specifically the CD14^+^CD16^+^ subset. Correspondingly, CD14^+^CD16^+^ monocytes/macrophages massively accumulate in the fibrotic/cirrhotic liver. Monocyte-related chemokine pathways are differentially activated in the liver and circulation of patients with liver disease. Functionally, CD14^+^CD16^+^ monocytes likely perpetuate intrahepatic inflammation via secretion of proinflammatory cytokines, but also directly activate profibrogenic HSC.

## Results

### Blood monocytes increase in patients with chronic liver disease, are associated with disease progression and shift towards the ‘non-classical’ CD16^+^ monocyte subset

**Figure 1:**
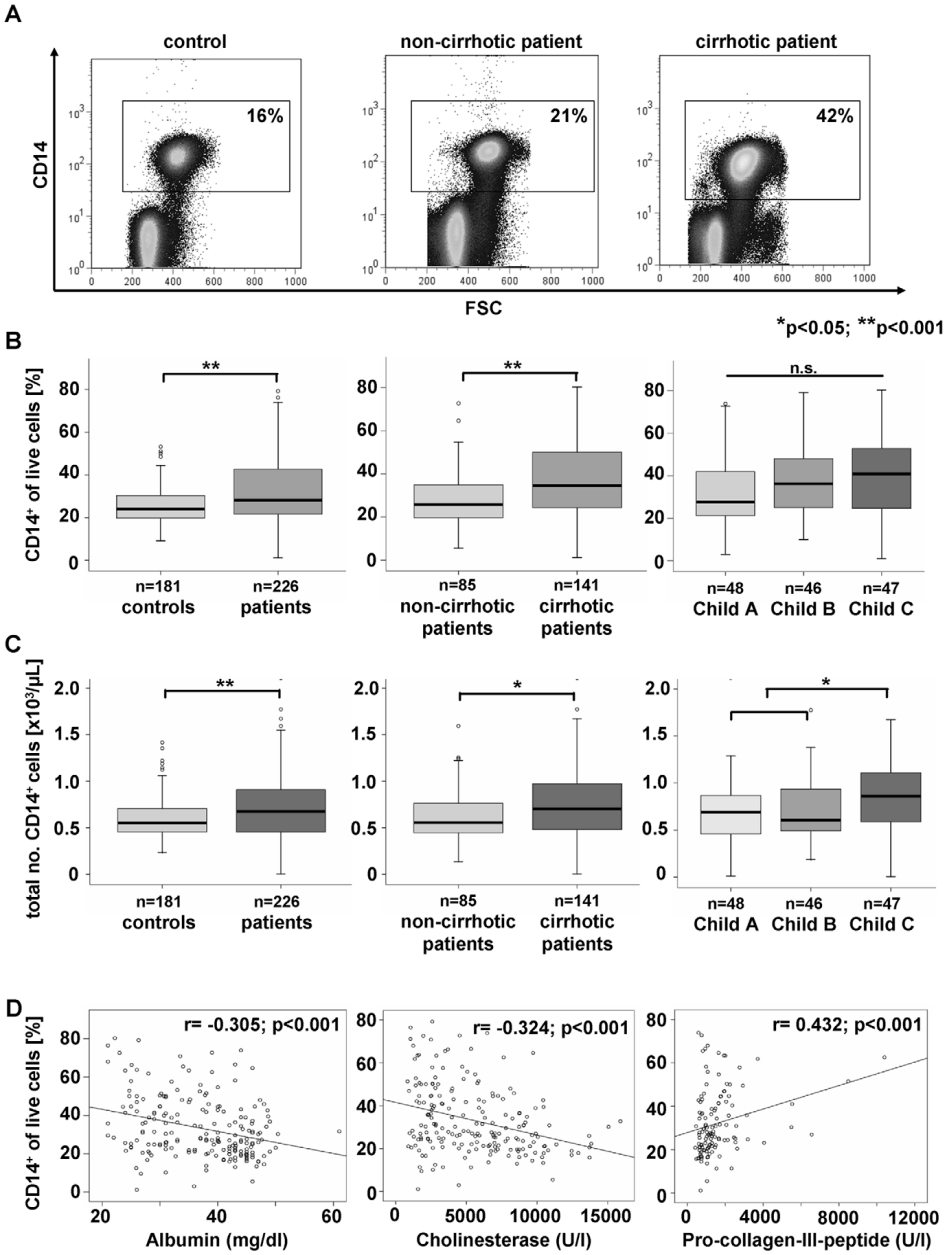
Blood monocytes increase in patients with chronic liver disease and are associated with disease progression. (A) Monocytes are defined by CD14 staining of PBMC (representative FACS plots shown). (B+C) Relative proportion of monocytes (CD14^+^) (B) and absolute monocyte numbers (C). (D) Association of circulating monocytes with laboratory parameters in CLD patients. *p<0.05, **p<0.001.

Recent reports from experimental liver injury in mouse models demonstrated an important functional role of the inflammatory Ly6C^hi^ (Gr1^hi^) monocyte subset for the progression of liver fibrosis, because the chemokine-driven accumulation of these monocyte-derived intrahepatic macrophages crucially perpetuates hepatic inflammation and can promote activation of hepatic stellate cells (HSC) as the main collagen-producing cells in the liver [6], [18]. In order to translate these findings from animal models into human pathogenesis, we subjected peripheral blood of 226 patients with chronic liver diseases (CLD) and 184 healthy controls to immediate FACS analysis. CLD patients had significantly higher circulating monocytes than controls, both as relative contribution to WBC (p = 0.002) as well as in absolute cell counts (p = 0.002, Fig. 1A–C, Table 1). Increasing monocyte numbers were associated with disease progression, specifically with the progression from non-cirrhotic to cirrhotic disease (Fig. 1A–C, Table 1). Patients with end-stage cirrhosis (Child C) showed higher blood monocytes than early stages of liver cirrhosis (p = 0.001, Fig. 1C). Moreover, there were inverse correlations between monocyte counts and parameters indicating the hepatic biosynthetic capacity, such as serum albumin (r = −0.305, p<0.001), prothrombin time (r = −0.310, p<0.001) or pseudocholinesterase activity (r = −0.324, p<0.001), and positive correlations to serological fibrosis markers, e.g. pro-collagen-III-peptide (r = 0.432, p<0.001) and hyaluronic acid (r = 0.241, p = 0.001, Fig. 1D, Table 2). Higher blood monocytes were also found in patients with clinical complications of CLD, such as icterus, encephalopathy, ascites or esophageal varices (data not shown). However, in patients with established liver cirrhosis, monocyte counts were not indicative of clinical complications (not shown).

**Table 1:** Characteristics of the patient cohort.

	Healthy controls	All patients	Stages of liver cirrhosis			
			No cirrh.	Child A	Child B	Child C
*n*	184	226	85	48	46	47
Sex (male/female) *n*	109/75	142/84	53/32	26/22	26/20	37/10
Age *yrs*	43 (16–68)	53 (17–82)	43 (17–73)	63 (30–82)	60 (28–77)	53 (21–81)
Liver disease etiology *n*	n.a.					
Viral hepatitis		89	49	19	15	6
Biliary/autoimmune		27	15	7	3	2
Alcohol		65	5	14	17	29
Other origin		45	16	8	11	10
Clinical complications *n*	n.a.					
Esophageal varices		85	0	21	29	35
Ascites		80	1	7	31	41
HCC		23	0	9	8	6
WBC *x10^3^/µl*	5.9 (1.7–11.6)	6.1 (1.4–28.8)	5.8 (2.2–14.3)	6.3 (2.0–22.3)	5.3 (1.8–16.3)	6.85 (1.4–28.8)
Total monocytes *x10^3^/µl*	0.55 (0.23–1.42)	0.68 (0.01–2.72)	0.60 (0.14–1.59)	0.69 (0.01–2.62)	0.61 (0.19–2.72)	0.86 (0.01–1.67)
CD14^+^CD16^−^ monocytes %	92.4 (78.2–97.9)	90.0 (72.1–98.7)	90.7 (78.2–98.7)	89.5 (72.1–97.6)	90.6 (77.2–97.1)	88.4 (77.1–98.7)
CD14^+^CD16^−^ monocytes *x10^3^/µl*	0.50 (0.2–1.31)	0.61 (0.01–2.55)	0.54 (0.12–1.57)	0.59 (0.01–2.55)	0.57 (0.17–2.54)	0.74 (0.01–1.47)
CD14^+^CD16^+^ monocytes %	7.5 (2.1–21.9)	9.9 (1.1–27.4)	9.3 (1.1–21.7)	10.7 (2.5–27.4)	9.4 (2.9–23.0)	11.2 (1.3–22.9)
CD14^+^CD16^+^ monocytes *x10^3^/µl*	0.04 (0.01–0.14)	0.06 (<0.01–0.5)	0.05 (0.01–0.17)	0.05 (<0.01–0.28)	0.06 (0.02–0.5)	0.09 (<0.01–0.38)
Serum MCP-1 *pg/ml* [CCL2]	88.3 (<1.3–237.0)	102.8 (<1.3–24794)	115.8 (16.8–365.2)	116.7 (17.2–24794)	90.3 (<1.3–904.8)	56.0 (<1.3–1484.5)
Serum MIP1α *pg/ml* [CCL3]	<1.3 (<1.3–2.8)	3.0 (<1.3–183.3)	2.2 (<1.3–60)	3.9 (<1.3–60)	4.5 (1.8–66.3)	3.4 (<1.3–183.3)
Serum MIP1(pg/ml [CCL4]	30.6 (<1.3–62.5)	47.2 (<1.3–443.9)	47.0 (<1.3–139.5)	53.7 (20.3–249.5)	46.7 (8.8–443.9)	41.6 (9.3–443.5)

For quantitative variables, the median is given with the range in parenthesis. n.a., not applicable; HCC, hepatocellular carcinoma; WBC, white blood cell count. For chemokine serum concentrations, alternative names are given in square brackets.

**Table 2:** Correlation analysis.

	total monocytes		CD14^+^CD16^−^ monocytes		CD14^+^CD16^+^ monocytes	
	R	*p*	r	*p*	r	*p*
**Clinical scores**						
Child-Pugh (points)	0.188	*0.038*	-	*n.s.*	-	*n.s.*
MELD	0.190	*0.031*	-	*n.s.*	-	*n.s.*
**Liver function**						
Bilirubin total	0.242	*0.000*	−0.202	*0.004*	0.174	*0.013*
Bilirubin conjugated	0.235	*0.001*	−0.218	*0.002*	-	*n.s.*
Albumin	−0.305	*<0.001*	−0.179	*0.011*	-	*n.s.*
PCHE	−0.324	*<0.001*	0.176	*0.013*	−0.174	*0.014*
Prothrombin time (%)	−0.310	*<0.001*	-	*n.s.*	−0.223	*0.001*
INR	0.264	*<0.001*	-	*n.s.*	0.167	*0.016*
Factor V	−0.172	*0.023*	-	*n.s.*	-	*n.s.*
**Fibrosis markers**						
Procollagen-III-peptide	0.432	*<0.001*	−0.315	*<0.001*	0.307	*<0.001*
Hyaluronic acid	0.241	*0.001*	-	*n.s.*	-	*n.s.*
**Inflammatory cytokines & chemokines**						
IL6	0.261	*<0.001*	-	*n.s.*	-	*n.s.*
TNFα	0.389	*<0.001*	−0.252	*0.001*	0.243	*0.002*
MCP-1 (CCL2)	-	*n.s.*	-	*n.s.*	0.261	*0.002*
MIP1β (CCL4)	-	*n.s.*	-	*n.s.*	0.150	*0.043*
MIG (CXCL9)	-	*n.s.*	−0.265	*0.002*	-	*n.s.*
IP-10 (CXCL10)	0.212	*0.018*	−0.244	*0.006*	0.169	*0.002*
**Hematology**						
Total WBC	0.282	*<0.001*	-	*n.s.*	-	*n.s.*
Lymphocyte count	−0.635	*<0.001*	-	*n.s.*	-	*n.s.*
Platelets	-	*n.s.*	-	*n.s.*	−0.186	*0.007*
Hemoglobin	-	*n.s.*	0.161	*0.020*	-	*n.s.*

Correlation analysis (Spearman rank correlation test) between total monocytes, the relative abundance of CD14^+^CD16^−^ or CD14^+^CD16^+^ monocytes and clinical scores, serum markers of liver function, inflammatory cytokine and chemokine serum concentrations and other blood counts are given in the table. Only significant results are shown. MELD, model of end stage liver disease; PCHE, pseudocholinesterase; INR, international normalized ratio; IL, interleukin; WBC, white blood cell count; *n.s.*, not significant.

**Figure 2:**
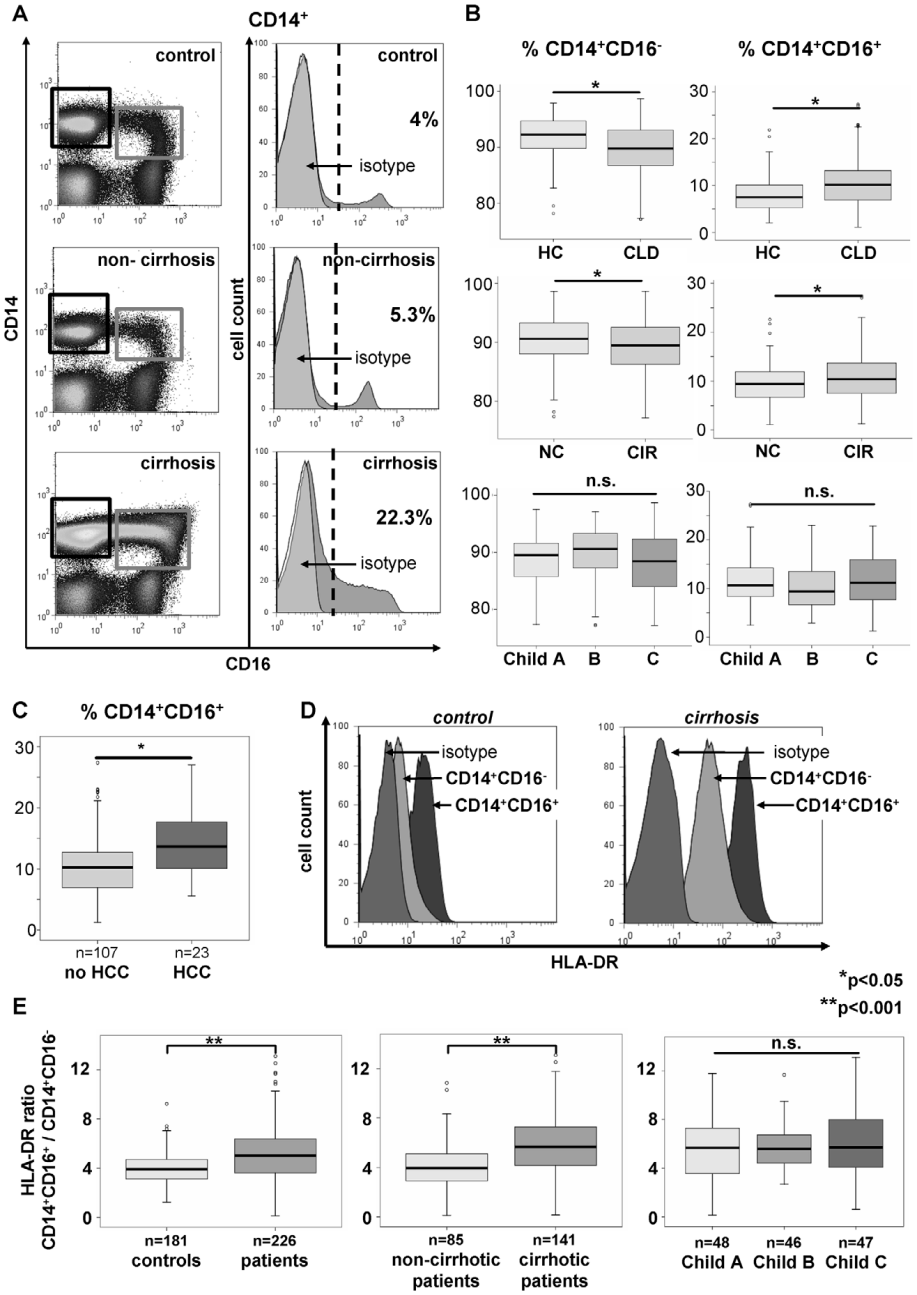
Relative increase of CD14^+^CD16^+^ blood monocytes and more activated phenotype in patients with liver cirrhosis. (A) Representative FACS plots displaying an increase of CD14^+^CD16^+^ monocytes (black gate: CD14^+^CD16^−^, grey gate: CD14^+^CD16^+^) among PBMC in patients with cirrhosis compared to healthy controls and non-cirrhotic patients (left). The histograms show the relative distribution of CD16 expression on CD14^+^ cells (right; grey: isotype control). (B) Statistical analysis of monocyte subsets. HC, healthy controls (n = 181); CLD, patients (n = 226); NC, non-cirrhotic (n = 85); CIR, cirrhotic (n = 141). (C) Patients with liver cirrhosis and hepatocellular carcinoma (HCC) have significantly (p = 0.008) higher CD16^+^ monocytes than cirrhotics without HCC. (D) Representative FACS staining for HLA-DR on monocyte subsets. (E) Ratio of HLA-DR expression on CD16^+^ vs. CD16^−^ monocytes. *p<0.05, **p<0.001.

In humans, the ‘classical’ CD14^+^CD16^−^ monocytes share many characteristics with murine Gr1^hi^ (Ly6C^hi^) monocytes, whereas ‘non-classical’ CD14^+^CD16^+^ cells are considered counterparts of murine Gr1^lo^ (Ly6C^lo^) monocytes [13]. The CD14^+^CD16^+^ subset has long been thought to constitute ‘inflammatory monocytes’ in humans [12]. Strikingly, we observed a strong shift towards the CD14^+^CD16^+^ monocyte subset in CLD patients, especially in patients with established cirrhosis (Fig. 2A–B). The increase in absolute numbers of both subsets, however, did not reach statistical significance (Fig. S1). The relative abundance of CD14^+^CD16^+^ monocytes was correlated with inflammatory cytokines and parameters indicative of disease progression, while CD14^+^CD16^−^ monocytes showed inverse correlations to these markers (Table 2), indicating a contribution of CD14^+^CD16^+^ monocytes to the chronic inflammatory state of patients with CLD and cirrhosis. Of note, we could not observe any differences in monocyte counts or monocyte subsets between the different underlying etiologies of CLD (data not shown), suggesting that the quantitative and qualitative changes in the monocyte compartment represent a rather uniform response during CLD progression and fibrogenesis. However, patients with liver cirrhosis and hepatocellular carcinoma (HCC) even displayed significantly higher CD14^+^CD16^+^ monocytes than cirrhotic patients without HCC (p = 0.008, Fig. 2C).

A striking feature of the two major monocyte subpopulations is their differential expression of the MHC-II molecule HLA-DR [13], because CD14^+^CD16^+^ express HLA-DR much stronger than the CD14^+^CD16^−^ cells (Fig. 2D). In CLD patients, HLA-DR expression is significantly upregulated on CD14^+^CD16^+^ monocytes (p = 0.027, Fig. 2D and Fig. S2), thereby indicating a markedly enhanced activation and maturation status. This results in a significant increase in the ratio of HLA-DR expression between both subsets in CLD patients and especially in those with liver cirrhosis (p<0.001, Fig. 2E). Collectively, these data demonstrate a substantial shift of circulating monocytes towards the ‘non-classical’ monocyte subset that is associated with inflammation, fibrosis and disease progression in CLD patients.

### Intrahepatic CD16^+^ macrophages predominantly increase during liver fibrosis progression

**Figure 3:**
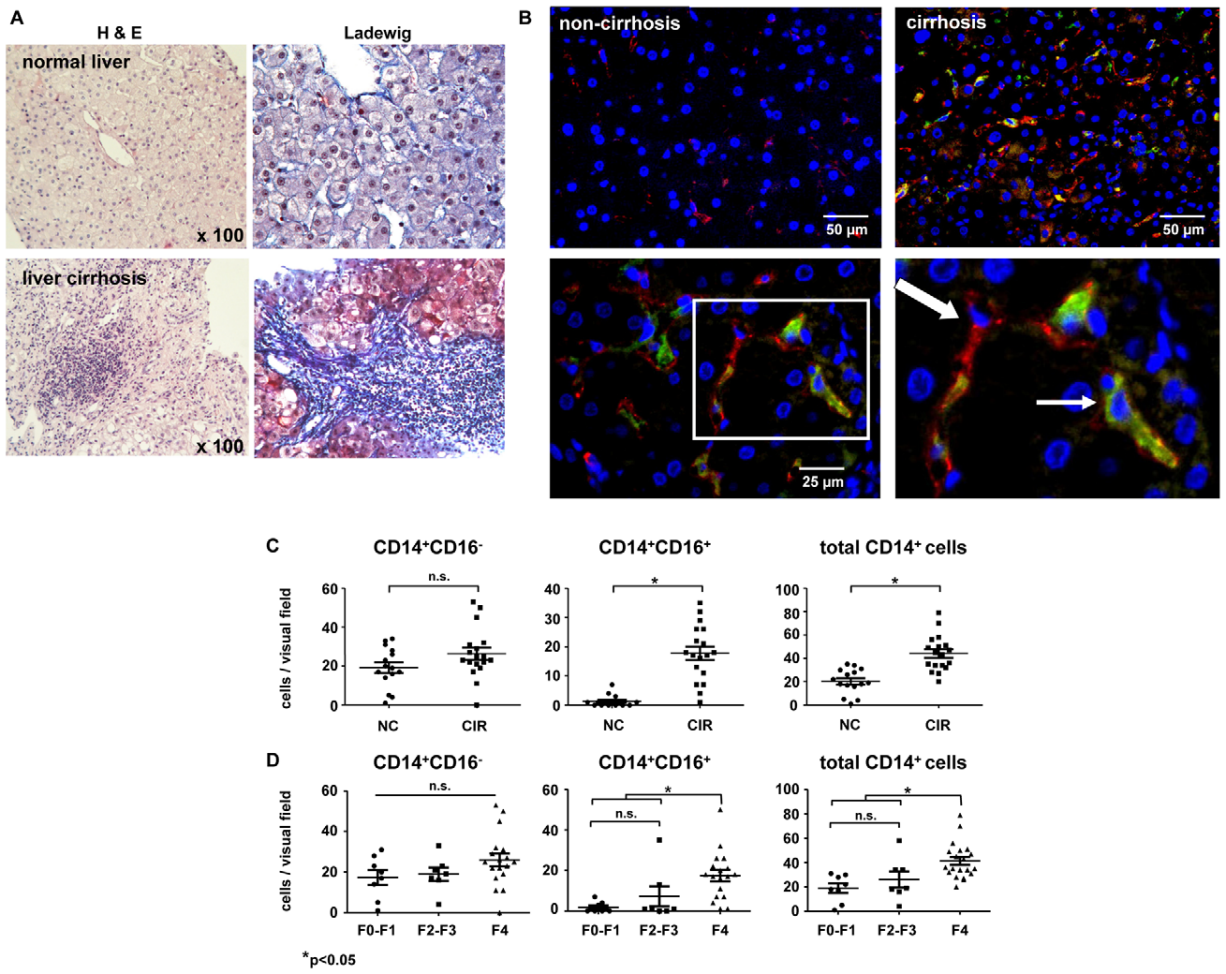
Intrahepatic CD16^+^ macrophages predominantly increase during liver fibrosis progression. (A) Representative examples of biopsies from normal liver (upper panel) and cirrhotic liver (lower panel) show mononuclear inflammatory infiltrates in fibrotic periportal regions (left: H&E staining, right: Ladewig staining, in which collagen stains blue). (B) Immunofluoresecent co-staining for CD14 (red) and CD16 (green) identifies CD14^+^CD16^−^ and CD14^+^CD16^+^ macrophages in human liver tissue (blue: nuclei counterstained with DAPI). Bold arrow, CD14^+^CD16^−^ macrophage; thin arrow, CD14^+^CD16^+^ macrophage. (C+D) Semiquantative analysis of CD14^+^CD16^−^, CD14^+^CD16^+^ and total CD14^+^ intrahepatic cells. *p<0.05, **p<0.001.

It is well established that the number of macrophages increases during chronic liver injury and fibrogenesis [4], but the phenotype of intrahepatic monocyte-derived cells remains poorly defined. Thus, we tested whether CD14^+^CD16^−^ and CD14^+^CD16^+^ monocyte/macrophage subpopulations are also present within the liver. In fact, conventional histology already identified mononuclear infiltrates in the portal regions of cirrhotic versus normal liver (Fig. 3A), and immunohistochemical co-staining for CD14 and CD16 was established to classify the intrahepatic monocytes/macrophages (Fig. 3B). We observed a significant increase of CD14^+^CD16^+^ cells in cirrhosis (p = 0.02 compared to F2–F3 fibrosis, p = 0.001 compared to F0–F1), which account for approximately 50% of the total intrahepatic monocytes/macrophages in cirrhotic, but only for about 10% in non-cirrhotic livers, and mostly explain the total increase in hepatic macrophages in cirrhosis (Fig. 3C). A similar trend was noticed when early stages of fibrosis (scored F0 and F1 by a blinded pathologist) were compared to progressive (F2–F3) and cirrhotic (F4) disease (Fig. 3D).

**Figure 4:**
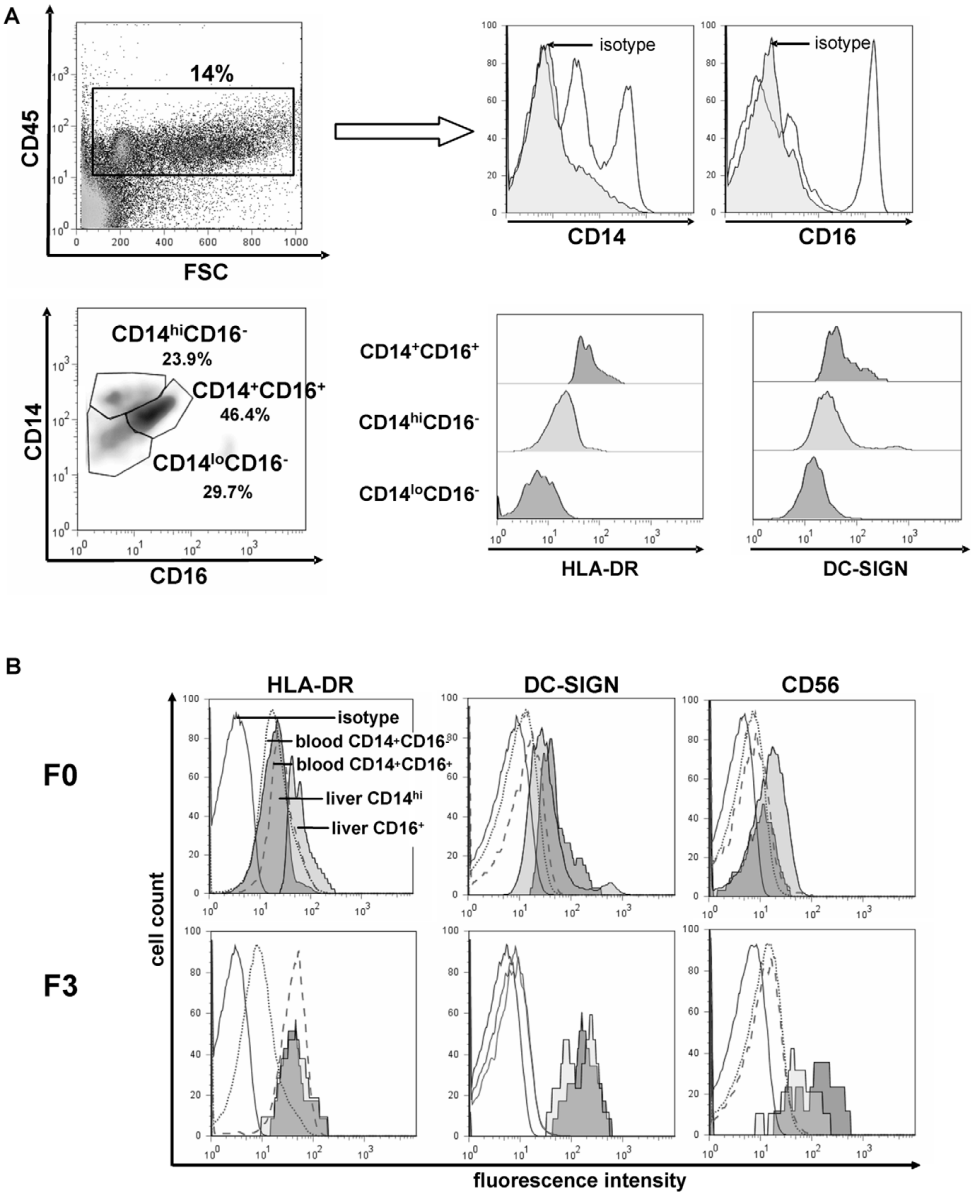
Intrahepatic macrophages consist of different subpopulations mirroring blood monocyte subsets. (A) FACS analysis of intrahepatic monocytes/macrophages, based on >30 fresh liver biopsies. Representative plots are displayed. Among the CD45^+^ intrahepatic leukocytes, three different populations of intrahepatic CD14^+^ macrophages can be distinguished based on CD14 and CD16 expression that also differ characteristically in HLA-DR and DC-SIGN expression. (B) Expression levels of monocyte/macrophage activation and differentiation markers were compared in the same patients between blood CD14^+^CD16^−^ monocytes (dotted line) and liver CD14^hi^CD16^−^ macrophages (dark grey) as well as CD14^+^CD16^+^ monocytes (dashed line) and liver CD14^+^CD16^+^ macrophages (light grey); representative analyses from patients with a F0 fibrosis (no fibrosis, upper panel) and a F3 fibrosis (advanced fibrosis, lower panel) are shown. Isotype control, black line. Blood was drawn at the time of liver biopsy, and blood/liver samples were run with the same FACS settings at the same time.

We next aimed to characterize these intrahepatic macrophage subpopulations further and to establish the relationship between intrahepatic and peripheral blood monocyte/macrophage subsets. Unlike in peripheral blood (Fig. 2A), FACS analysis from freshly obtained liver biopsies (n>30) revealed the existence of three CD14^+^ intrahepatic monocyte/macrophage populations (Fig. 4A) that could be defined as CD14 high-expressing cells (CD14^hi^CD16^−^), CD14 low-expressing cells and CD14/CD16 double-positive macrophages (CD14^+^CD16^+^). Characteristically, CD14^hi^CD16^−^ hepatic cells expressed low HLA-DR and low DC-SIGN (CD209), similar to peripheral CD14^+^CD16^−^ monocytes. In contrast, the intrahepatic CD14^+^CD16^+^ cells expressed high HLA-DR and some DC-SIGN (CD209), similar to peripheral CD14^+^CD16^+^ monocytes (Fig. 4A). In addition, we found CD14^lo^ cells which are negative for CD16, HLA-DR and DC-SIGN, hence likely representing sessile hepatic macrophages (classical Kupffer cells).

In order to further substantiate these observations, intrahepatic macrophage subsets were directly compared to peripheral blood monocyte subpopulations in representative patients. Despite the principal similarities between intrahepatic CD14^+^CD16^+^ and circulating CD14^+^CD16^+^ cells, intrahepatic CD16^+^ macrophages showed up-regulated HLA-DR, DC-SIGN and (moderately) CD56 expression in comparison to their blood counterparts (Fig. 4B), thereby indicating intrahepatic maturation of this macrophage population. The CD14^hi^CD16^−^ cells, on the other hand, express similar levels of HLA-DR as circulating CD14^+^CD16^−^ monocytes, but differ by displaying higher levels of the differentiation markers DC-SIGN and CD56 (Fig. 4B). Interestingly, HLA-DR expression appeared down-regulated on CD14^+^CD16^+^ intrahepatic macrophages in patients with advanced compared to early or absent fibrosis, while DC-SIGN was up-regulated, further indicating an activated (pro-inflammatory) state of this macrophage subpopulation in advanced fibrosis (Fig. 4B).

### Activation of monocyte-related chemokine pathways in chronic liver disease

**Figure 5:**
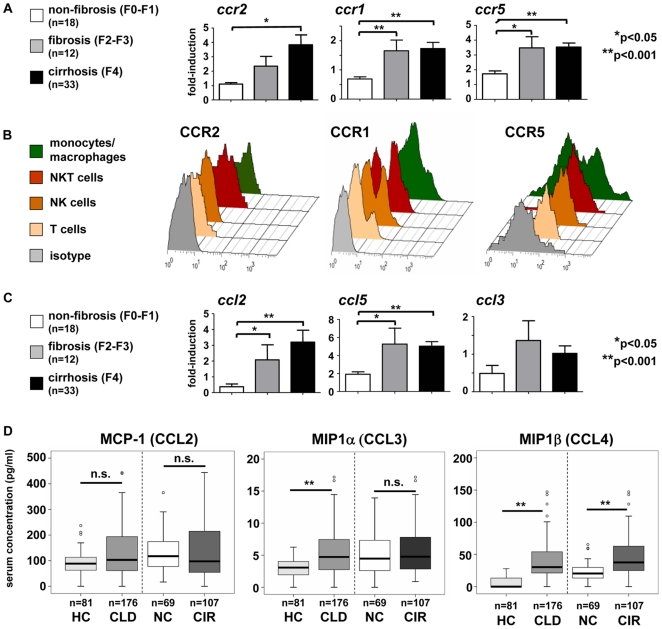
Activation of monocyte-related chemokine pathways and of monocytic chemokine receptors in chronic liver disease. (A) Intrahepatic gene expression levels of chemokine receptors. (B) Expression of CCR2, CCR1 and CCR5 was assessed by FACS on monocytes/macrophages (CD14^+^, green), T- (CD3^+^CD56^−^, light orange), NK- (CD3^−^CD56^+^, dark orange) and NKT-cells (CD3^+^CD56^+^, red) from freshly isolated liver tissue. Representative histograms are shown, isotype control in grey. (C) Intrahepatic gene expression levels of chemokines. (D) Serum concentrations of monocyte-related chemokines in patients with chronic liver diseases and healthy controls. Abbreviations are: HC, healthy control; CLD, chronic liver disease; NC, no cirrhosis; CIR, cirrhosis. *p<0.05, **p<0.001.

Our data demonstrate the distinct accumulation of CD16^+^ monocytes in the liver during fibrosis progression, prompting us to study possible chemokine pathways that are activated in CLD and could mediate monocyte subset infiltration. In experimental murine models, the chemokine receptors CCR2, CCR1 or CCR5 that are differentially expressed on monocyte subsets have been implicated in hepatic fibrosis progression [6], [8], [11]. In humans, CCR2 is primarily expressed on CD14^+^CD16^−^ monocytes, whereas CD14^+^CD16^+^ monocytes express higher levels of CCR5 on their surface; CCR1 is expressed on both subsets, with moderately higher levels on CD14^+^CD16^−^ monocytes [13], [19], [20]. Gene expression analysis from whole liver tissue at different stages of fibrosis progression demonstrated a clear up-regulation of intrahepatic *ccr2* (F0-1 compared to F4 fibrosis, p = 0.021), *ccr5* (F0-1 compared to F4 fibrosis, p<0.0001) and *ccr1* (F0-1 compared to F4 fibrosis, p = 0.0008) in fibrosis (Fig. 5A), which matches well with the observed accumulation of monocytes in the fibrotic/cirrhotic liver (Fig. 3). As not only monocytes/macrophages, but also other immune cell subsets or non-parenchymal liver cells may express these chemokine receptors [2], we performed FACS analyses from fresh liver samples after biopsy and surgical resection. CCR2 expression was primarily found on hepatic monocytes/macrophages (defined as CD14^+^ cells) and (at lower levels) on intrahepatic NKT-, but not NK- or T-cells (Fig. 5B). CCR1 was expressed at high levels by almost all CD14^+^ cells, but also by subsets of T-, NK- and NKT-cells (Fig. 5B). CCR5 expression was primarily found on T-cells and subsets of NK- and NKT-cells, but hepatic monocytes/macrophages also express CCR5 at variable levels (Fig. 5B).

In line, hepatic mRNA expression of the chemokines *ccl2* (F0-1 compared to F4 fibrosis, p = 0.0088) and *ccl5* (F0-1 compared to F4 fibrosis, p<0.0001), but not of *ccl3*, was strongly up-regulated in fibrosis (Fig. 5C). Moreover, the serum concentrations of the CCR1/CCR5 ligands MIP1α (CCL3) and MIP1β (CCL4), but not of the CCR2 ligand MCP-1 (CCL2), were significantly increased in CLD patients (Fig. 5D), suggesting additional systemic actions of these chemokines.

**Figure 6:**
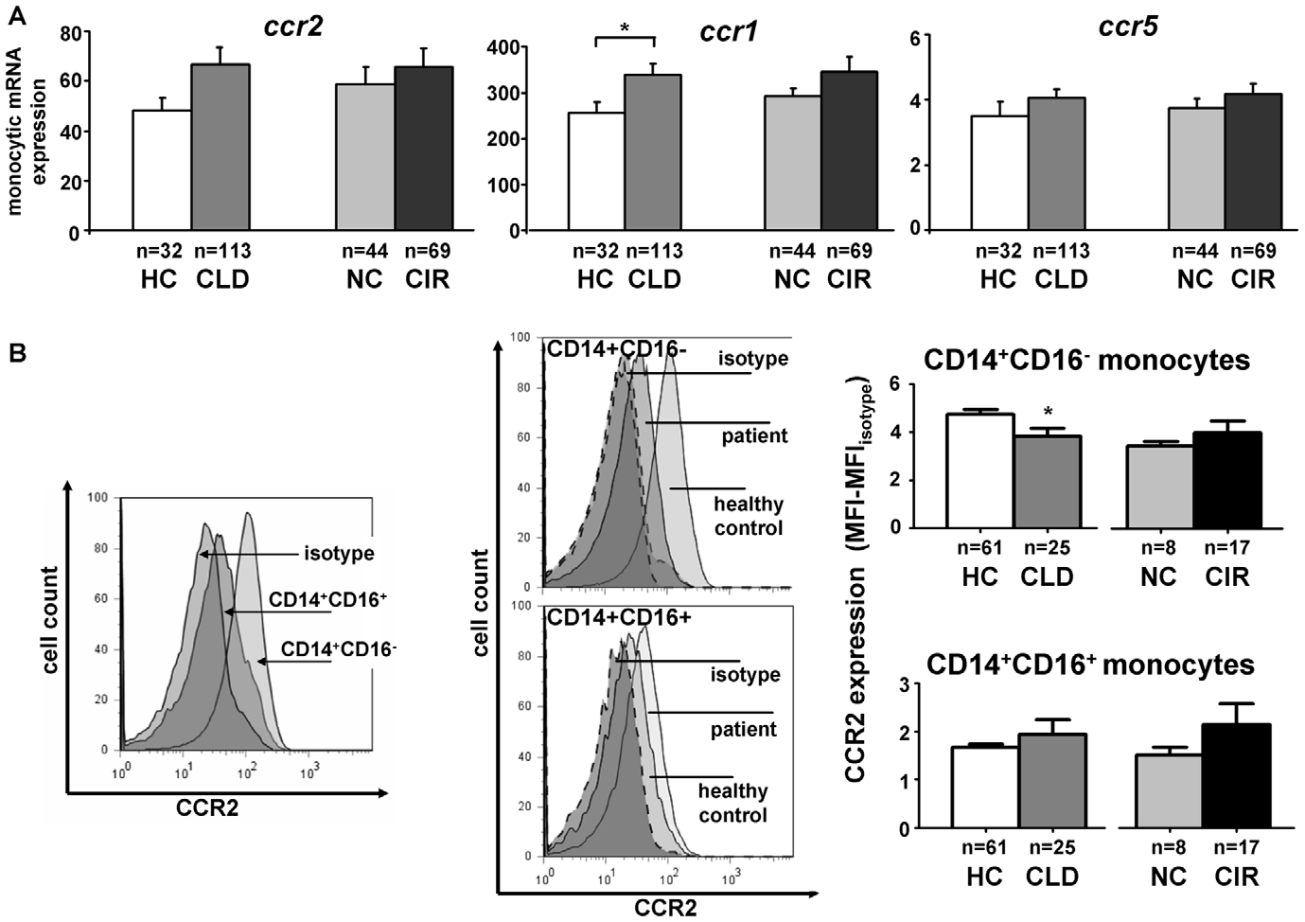
Regulation of chemokine receptors on circulating monocytes in chronic liver disease. (A) Monocytic chemokine receptor gene expression by real-time PCR after purification of circulating monocytes by CD14 microbeads (MACS). (B) CCR2 expression (MFI, mean fluorescence intensity) on blood monocyte subsets by FACS. Abbreviations are: HC, healthy control; CLD, chronic liver disease; NC, no cirrhosis; CIR, cirrhosis. Representative histograms are shown, comparing either CCR2 expression levels between both monocyte subsets as well as between healthy controls and CLD patients on the two monocyte subpopulations in peripheral blood. *p<0.05, **p<0.001.

Given the expression of CCR2, CCR1 and CCR5 by hepatic monocytes/macrophages, the local upregulation of *ccl2* and *ccl5* in the whole liver and the systemic elevation of CCL3 (healthy controls compared to CLD patients, p = 0.0387) and CCL4 (healthy controls compared to CLD patients, p = 0.0064) in the circulation, we speculated that peripheral blood monocytes in patients might regulate their chemokine receptor expression, rendering them more prone to accumulate in the diseased liver. We therefore isolated circulating monocytes by CD14-microbeads via MACS methodology from patients (n = 113) and healthy controls (n = 32) at purities greater than 95% (not shown). Monocytic *ccr1* (p = 0.031 for healthy controls compared to CLD patients), but not *ccr2* or *ccr5*, expression was increased on peripheral monocytes in patients (Fig. 6A), possibly in response to elevated serum levels of CCL3 and CCL4. With respect to monocyte subsets, CCR2 was predominantly expressed on CD14^+^CD16^−^ monocytes (Fig. 6B). On a protein level, CCR2 surface expression was modestly down-regulated on CD14^+^CD16^−^ monocytes of patients as compared to healthy controls, but not on CD14^+^CD16^+^ cells (Fig. 6B). Within the liver, CD14^hi^CD16^−^ macrophages expressed CCR2 at similar (high) levels as circulating CD14^+^CD16^−^ monocytes; liver CD14^+^CD16^+^ macrophages, in contrast, displayed lower levels than CD14^hi^CD16^−^ macrophages, but higher CCR2 expression than blood CD14^+^CD16^+^ monocytes, suggesting that the CD14^+^CD16^+^ subset up-regulated CCR2 intrahepatically (detailed data not shown). These results collectively revealed that monocyte-related chemokines targeting CCR2 and CCR1/CCR5 are up-regulated in the intra- and extrahepatic compartment of CLD patients.

### Functionality of monocytes in liver cirrhosis and differential cytokine and chemokine secretion by monocyte subsets

**Figure 7:**
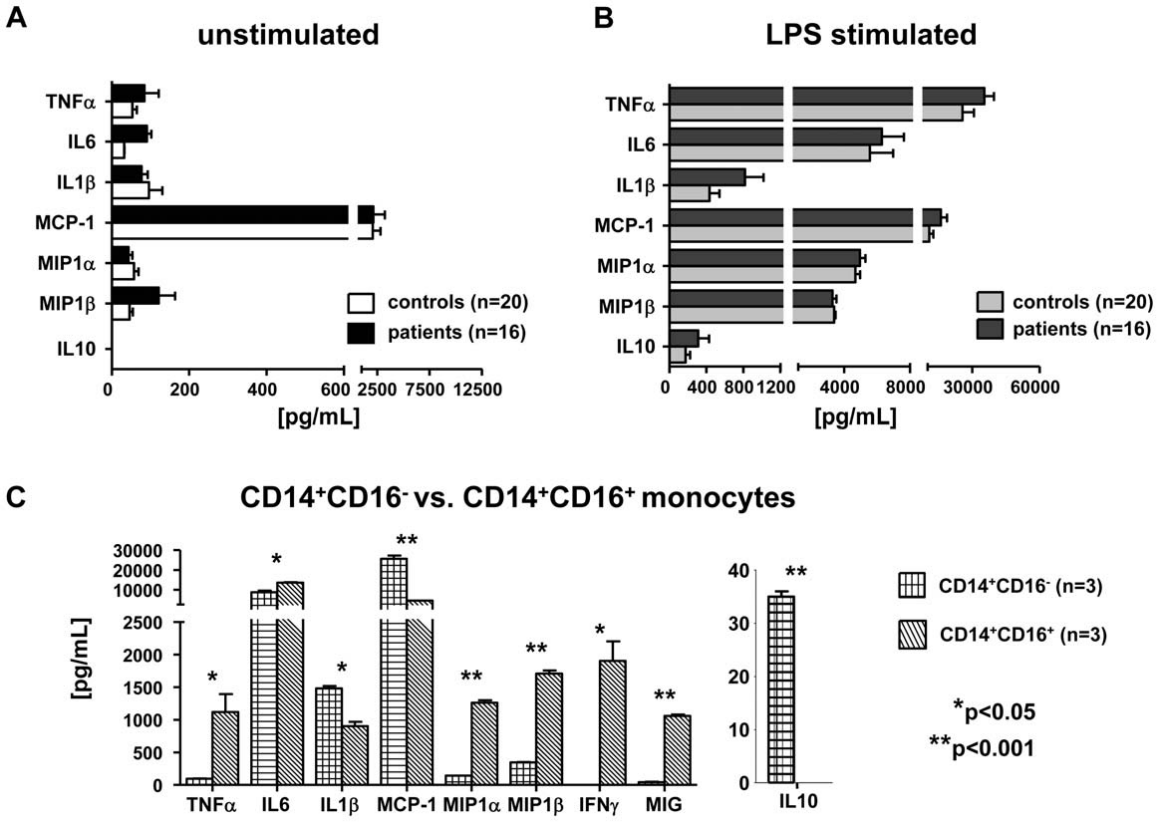
Monocytes are functionally active in liver cirrhosis with a differential release of distinct cytokines/chemokines by monocyte subsets. (A+B) Cytokine/chemokine release of monocyte-derived macrophages (2 days in culture) without stimulation (A) and after stimulation with 1 mg/ml LPS (B). (C) Cytokine/chemokine release of purified monocyte subsets after 5 days of culture without stimulation. *p<0.05, **p<0.001.

Although we consistently found more circulating monocytes in CLD patients and a close association with disease progression (Fig. 1, Table 2), it remained unclear if these monocytes were fully functionally active. It had been speculated before that monocyte activation might be impaired in liver cirrhosis, contributing to a so-called “immune-paralysis” in those patients [21], [22]. We therefore cultured isolated circulating monocytes from patients with advanced liver cirrhosis (Child B/C, n = 16) and matched healthy controls (n = 20) in media supplemented with autologous serum and assessed the secretion of proinflammatory cytokines and chemokines after LPS stimulation. Monocytes from healthy volunteers secreted high amounts of proinflammatory cytokines (TNFα, IL6, IL1β) and chemokines (MCP-1, MIP1α, MIP1β) upon stimulation with LPS (Fig. 7A–B). The chemokine MIG was not significantly induced by LPS. Monocytes isolated from patients with advanced cirrhosis did not differ with respect to any of the cytokines or chemokines analyzed at baseline or after LPS stimulation (Fig. 7A–B), suggesting that circulating monocytes in CLD patients preserved overall their normal capacity to secrete pro- or anti-inflammatory mediators.

Given the marked preferential intrahepatic accumulation of CD14^+^CD16^+^ monocytes in liver cirrhosis (Fig. 3), we next aimed to define the likely function of this subset in the pathogenesis of chronic liver inflammation and fibrosis. CD14^+^CD16^−^ and CD14^+^CD16^+^ monocytes were isolated by MACS methodology, and cytokine/chemokine secretion was measured after five days of culture without additional stimulation. Due to ethical considerations (large blood volume required for subset isolation) and based on the identical cytokine secretion of total monocytes upon stimulation (Fig. 7A–B), monocyte subpopulations were only isolated from healthy volunteers. Strikingly, CD14^+^CD16^+^ monocytes were the major producers of TNFα, IL6 (CD14^+^CD16^+^ vs. CD14^+^CD16^−^, p = 0.038), IFNγ (CD14^+^CD16^+^ vs. CD14^+^CD16^−^, p = 0.0242), MIP1α (CD14^+^CD16^+^ vs. CD14^+^CD16^−^, p = 0.0011) and MIP1β (Fig. 7C), indicating that they primarily perpetuate inflammatory processes by releasing proinflammatory cyto- and chemokines. This conclusion is corroborated by correlations between circulating CD14^+^CD16^+^ monocyte counts and proinflammatory serum cytokine levels (e.g., TNFα, MIP1β) in CLD patients (Table 2).

CD14^+^CD16^−^ monocytes, on the other hand, were the main producers of MCP-1 (CD14^+^CD16^+^ vs. CD14^+^CD16^−^, p = 0.0068), in line with observations that MCP-1 can stimulate MCP-1 expression via CCR2 binding in an autocrine manner [23]. Moreover, CD14^+^CD16^−^, but not CD14^+^CD16^+^ monocytes were capable of producing the antiinflammatory cytokine IL10 (Fig. 7C). Collectively, these data imply that the CD14^+^CD16^+^ monocytes that accumulate in the fibrotic/cirrhotic liver are important sources of proinflammatory mediators thereby perpetuating the chronic inflammation in the liver.

### CD16^+^ monocytes directly activate hepatic stellate cells

**Figure 8:**
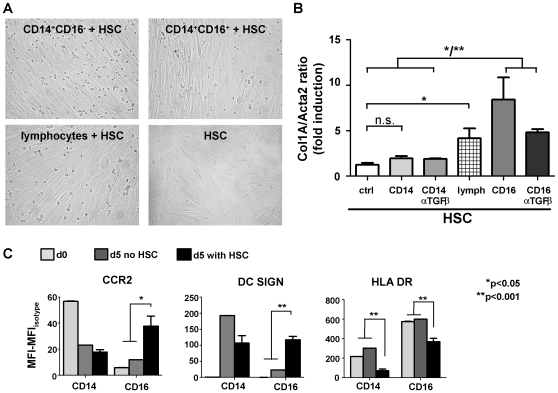
CD14^+^CD16^+^, but not CD14^+^CD16^−^ monocytes directly activate hepatic stellate cells. (A) Primary human HSC were isolated and co-cultured for 5 days with CD14^+^CD16^−^, CD14^+^CD16^+^ monocytes or lymphocytes. No morphological differences were noted on HSC in these conditions. (B) HSC activation was determined by *collagen-1A (col1A)* mRNA expression, normalized to the ‘HSC-house keeping gene’ *Acta2*. (C) Expression of surface molecules by FACS (MFI, mean fluorescence intensity) on CD14^+^CD16^−^ (CD14) and CD14^+^CD16^+^ (CD16) monocytes/macrophages at day 0, and after 5 days in culture or co-culture with HSC. All results derived from three independent experiments. *p<0.05, **p<0.001.

Monocyte-derived macrophages can activate HSC and hence are potent inductors of liver fibrosis [18]. In murine models of hepatic fibrosis, Gr1^+^ monocytes (‘classical monocytes’) can directly activate HSC in a TGFβ-dependent manner [6]. We therefore assessed possible effects of human monocyte subpopulations on HSC by co-culturing either subset with primary HSC isolated from explanted human livers of three independent donors (Fig. 8A). The experimental set-up was validated by stimulating primary HSC by recombinant TGFβ, which resulted in significant up-regulation of *col1A* mRNA, but not of *Acta2*; expression of *Acta2* could therefore be used as a house-keeping gene for HSC (not shown). In the co-culture experiments CD14^+^CD16^+^ monocytes, but not CD14^+^CD16^−^ monocytes significantly up-regulated collagen gene expression in HSC (CD14^+^CD16^+^ vs. CD14^+^CD16^−^, p = 0.0243) (Fig. 8B). It could be excluded that CD14^+^CD16^+^ monocytes directly differentiated into collagen-producing fibrocytes *in vitro* [24], because cultures of monocyte subsets by itself did not result in detectable collagen mRNA within five days (not shown). Of note, also the mixed population of lymphocytes induced HSC activation, highlighting that not only macrophages, but also NK, NKT and T cell subsets may interact with HSC during fibrosis development [2]. The activation of HSC by CD14^+^CD16^+^ monocytes could be partially blocked by anti-TGFβ antibodies (Fig. 8B). These data indicate that ‘non-classical’ CD14^+^CD16^+^ monocytes not only provide proinflammatory cytokines, but also exert direct fibrogenic actions on HSC.

Moreover, co-culture with HSC in turn differentially affected the expression of chemokine receptors and activation markers of monocyte subsets. CCR2 and DC-SIGN were strongly induced upon co-culture with HSC only on CD14^+^CD16^+^ monocytes (CCR2 expression at d0 compared to d5, p = 0.0147; DC-SIGN expression at d0 compared to d5, p = 0.0004) (Fig. 8C), indicating that the increase of *ccr2* mRNA transcripts in whole liver (Fig. 5A) might be partially attributed to up-regulated expression by CD16^+^ monocyte-derived macrophages. In contrast, HLA-DR was down-regulated in response to co-culture with HSC in both monocyte subsets (Fig. 8C). These observations emphasize that monocytes/macrophages distinctly interact with other cellular components of the hepatic microenvironment.

## Discussion

Accumulating evidence from murine models indicated that monocyte infiltration into the liver is a major pathogenic factor for chronic hepatic inflammation and fibrosis [5], [6], [7], [8]. In this study, we demonstrate that monocytes increase in the circulation as well as in the liver of patients during progression of chronic liver disease, and that this is associated with a shift towards the ‘non-classical’ subset of CD14^+^CD16^+^ monocytes. These CD14^+^CD16^+^ cells have an activated phenotype and produce high amounts of pro-inflammatory cytokines and chemokines upon differentiation. Given the assumption that CD14^+^CD16^+^ monocytes would resemble Gr1^lo^ cells in mice, our findings reveal a considerable discrepancy from mouse models, because fibrosis induction and progression in mice is accompanied by Gr1^hi^ monocytosis in peripheral blood and infiltration of Gr1^hi^ monocytes into the injured liver [6], [7]. One obvious difference between murine models and the human diseased liver is the strikingly dissimilar time-course of fibrosis development. Whereas experimental murine fibrosis is analyzed at three or six weeks after induction, e.g. by biliary duct ligation or carbon tetrachloride injection, human fibrosis and cirrhosis usually develops over decades of chronic injury and inflammation. Human cirrhosis is thereby a more advanced disease with respect to collagen deposition, tissue reorganization and myofibroblast activation than even 8-weeks-murine fibrosis models [25]. In this respect, it is important to point out that the most prominent enrichment of these non-classical CD14^+^CD16^+^ monocytes in peripheral blood and in the liver was observed in patients with liver cirrhosis, in contrast to the similar levels observed between healthy volunteers and CLD patients at early stages of liver fibrosis. This suggests that the proposed pro-inflammatory and profibrogenic actions of CD14^+^CD16^+^ monocytes/macrophages are most relevant at advanced fibrosis or cirrhosis, possibly explaining different observations between human cirrhosis and experimental mouse models.

The assumption that CD14^+^CD16^+^ human monocytes are equivalents of murine Gr1^lo^ monocytes is primarily based on conserved gene and protein profiles between these subsets [13], but not on functional assays. In fact, murine Gr1^hi^ monocyte-derived cells in inflammatory conditions and human CD14^+^CD16^+^-derived macrophages share important functional properties, particularly the expression of pro-inflammatory cytokines such as TNFα or nitric oxide [3], [26]. Our study revealed correlations between CD14^+^CD16^+^ monocytes and pro-inflammatory cytokines/chemokines in patients as well as a preferential secretion of pro-inflammatory cytokines by this subset, suggesting that the increase of CD14^+^CD16^+^ monocytes in patients with liver cirrhosis as the ‘inflammatory monocyte subset’ thereby mirrors the increase of Gr1^hi^ monocytes in murine models.

This raised the question if similar chemokine-pathways are activated in human liver diseases as in murine experimental models, given the substantial differences in chemokine receptor expression between murine Gr1^hi^ and human CD14^+^CD16^+^ monocytes [12]. Several recent independent animal studies defined an important function of CCR2 and MCP-1/CCL2 for hepatic fibrosis [5], [6], [7], [8]. In analogy to these findings, upregulated intrahepatic MCP-1 expression has been described during human hepatic fibrogenesis, predominantly by HSC, biliary epithelial cells and macrophages, and directly correlated with the number of hepatic macrophages in a small group of 15 patients [27]. We confirmed these observations in our large cohort, as *ccl2* and also *ccr2* mRNA transcripts were significantly increased in cirrhotic livers. However, unlike in mice where MCP-1 is thought to promote the exit of Gr1^hi^ monocytes from the bone marrow into the circulation [6], [28], systemic levels of MCP-1 were not significantly regulated in liver disease patients in comparison to healthy controls. Moreover, CCR2 expression was moderately lower in CD14^+^CD16^−^ monocytes of patients compared to controls and slightly increased in CD14^+^CD16^+^ monocytes. This might possibly indicate distinct local functions of CCR2/MCP-1 interactions in the liver during fibrosis progression, likely not limited to CCR2^hi^-expressing CD14^+^CD16^−^, but also on CD14^+^CD16^+^ monocytes. This hypothesis is corroborated by the fact that CD14^+^CD16^+^, but not CD14^+^CD16^−^ monocytes strongly upregulate CCR2 expression upon co-culture with HSC. *In-vitro*-experiments suggested that MCP-1 may activate the expression of profibrogenic genes such as TGFβ or pro-α1 chain of type I collagen in monocyte-derived macrophages by an MCP-1/CCR2-dependent amplification loop [23], indicating that local intrahepatic MCP-1 may fulfil other functions in addition to regulating monocyte recruitment in liver cirrhosis.

On the other hand, CCR1- and CCR5-related chemokines might contribute to monocyte recruitment. It is well established that HSC express CCL5/RANTES upon activation [29], [30]. We found a clear induction of intrahepatic *ccl5* expression, confirming a smaller prior study including 15 patients [11], alongside elevated serum CCL3/MIP1α, CCL4/MIP1β and CCL5/RANTES (not shown) concentrations in patients versus controls. Moreover, monocytic *ccr1* expression, but not *ccr5*, was increased in patients. These data demonstrate that monocyte-related chemokine pathways targeting CCR2, CCR1 and CCR5 are activated in patients with liver cirrhosis, likely regulating recruitment (CCR1, CCR5, CCR2) and local differentiation/activation (CCR2) of monocyte subsets in patients with chronic liver diseases. However, it is important to note that CCR1 and CCR5 expression is not restricted to monocyte/macrophages, but also found on other immune cells subsets within the, namely T-, NK- and NKT-cell populations [2], [31]. Both CCR1 and CCR5 have also been described on other non-parenchymal liver cells, including resting and activated HSC [11], [30]. Therefore, elevation of circulating or intrahepatic CCL3-CCL5 chemokines likely not only influences monocyte/macrophage recruitment, but also other cell populations in the diseased liver.

In patients with liver cirrhosis, intrahepatic monocytes/macrophages are significantly increased [4], and our analysis revealed that this can be mainly attributed to a selective accumulation of CD14^+^CD16^+^ monocytes/macrophages in the cirrhotic liver. Cells of the monocytic lineage are important elements of the hepatic inflammation, because these cells can phagocytize foreign material, present antigen to T cells, and produce a host of cytokines, including TNFα, IL1 and IL6 [4]. Dissecting the diverse functional capacities of both monocyte subsets *in vitro* confirmed that the CD14^+^CD16^+^ monocyte subset is the main producer of pro-inflammatory cytokines and chemokines such as TNFα, IL6, IFNγ, MIP1α and MIP1β, while CD14^+^CD16^−^ monocytes readily secrete more MCP-1, IL1β and IL10 [12]. Moreover, in line with experiments co-culturing murine Gr1^hi^ monocytes and murine HSC [6], CD14^+^CD16^+^ monocytes were also able to directly activate primary human HSC upon co-culture. These data indicate that non-classical CD14^+^CD16^+^ monocytes are crucial regulators in the pathogenesis of CLD in humans by secreting an abundance of cytokines perpetuating chronic inflammatory processes within the liver and by directly activating HSC that in turn can secrete multiple chemokines for monocyte recruitment [18]. Our study furthermore suggests that the modulation of monocyte-subset recruitment into the liver and subsequent differentiation in the inflamed hepatic environment may represent possible novel approaches for interventions targeting proinflammatory and profibrogenic actions of either monocyte subset in chronic liver diseases and liver fibrosis.

## Methods

### Patients and controls

The study protocol was approved by the local ethics committee (ethics committee of University Hospital Aachen, RWTH Aachen), and written informed consent was obtained from each patient. The study was conducted according to the principles expressed in the Declaration of Helsinki. Inclusion criteria were either any CLD with a predisposition to liver fibrosis or an already established liver fibrosis/cirrhosis of any origin. Established cirrhosis (in contrast to non-cirrhotic CLD) was defined, if imaging (ultrasound, CT or MRI scan), biopsy or laparoscopy indicated liver cirrhosis or if cirrhosis-related complications were present. Patients with established liver cirrhosis were staged according to Child-Pugh's criteria [32]. Patients with acute liver failure or acute hepatitis B or C were not included. Exclusion criteria were conditions known to directly affect monocyte subset distributions in humans, specifically ongoing bacterial infections (procalcitonin concentration above normal value [<0.5 µg/L]), HIV-infection, systemic steroid medication (prednisolone >7.5 mg/d or equivalent doses) and malignant tumor(s) except hepatocellular or cholangiocellular carcinoma. Furthermore, patients were excluded in case of systemic inflammatory response syndrome (SIRS) or sepsis criteria [33]. The etiologies of liver diseases comprised viral hepatitis (n = 89, 39.4%; HBV n = 38, HCV n = 51), biliary or autoimmune disease (n = 27, 11.9%; autoimmune hepatitis n = 10, primary biliary cirrhosis n = 8, primary sclerosing cholangitis n = 9), alcoholic liver disease (n = 65, 28.7%) and other liver diseases (n = 45, 20%, e.g. non-alcoholic steatohepatitis n = 7, hemochromatosis n = 4, cryptogenic n = 23). Grading and staging of liver samples (biopsies and explants) were performed according to Desmet-Scheuer score by one experienced pathologist, who was fully blinded to any experimental data [34].

As a control group, 181 healthy volunteers were recruited from the local blood transfusion institute that had normal aminotransferase activities, no history of liver disease or alcohol abuse and tested negative for HBV, HCV and HIV infections.

### FACS analysis of circulating monocyte subsets and intrahepatic macrophages

Fresh blood samples were collected by venipuncture in the morning in EDTA separator tubes from all patients and controls and promptly applied to PBMC isolation by Ficoll density gradient, using LSM-1077 (PAA, Pasching, Austria) and standard protocols [13]. After blocking nonspecific binding, the following monoclonal antibodies and appropriate isotype controls were used: CD14, CD16, CD56, HLA-DR, CD3, CD4, CD8, CD56, CD209/DC-SIGN, CD19 and CD45 (all BD, Heidelberg, Germany); CCR2, CCR1, CCR5 (R&D Systems, Minneapolis, MN). Flow cytometric analysis was performed on a FACS-Canto-II (BD), data were analysed by FlowJo software (TreeStar, Ashland, OR). In order to exclude that difference in cell isolation procedures for FACS analysis influences cell counts, absolute numbers for circulating cells were calculated using the relative values from FACS and automated WBC counts without the PMN fraction. Cell surface marker expression was quantified by determining mean fluorescence intensity minus the respective isotype control (‘MFI-MFI_isotype_’). For flow cytometric characterization of intrahepatic monocytes, a small piece of fresh liver biopsy cylinders was minced in PBS and digested with collagenase type IV (Worthington, Lakewood, NJ) for 30 min at 37°C, and subjected to staining for FACS [6].

### Monocyte separation, RNA isolation and gene expression analysis

After isolation of PBMC by density gradient, total monocytes were purified using CD14-microbeads and MACS separation technique (Miltenyi, Bergisch Gladbach, Germany). FACS analysis confirmed a purity of >95%. RNA was isolated from purified blood monocytes by pegGOLD (peqLab, Erlangen, Germany), and complementary DNA was generated from 1 µg RNA (Roche, Mannheim, Germany). Quantitative real-time PCR was performed using SYBR Green Reagent (Invitrogen, Karlsruhe, Germany). β-actin values were used to normalize gene expression. Gene expression was either expressed by fold induction or arbitrary relative expression [14]. Primer sequences are available upon request. RNA and gene expression analyses from liver tissue, cell-culture and co-culture experiments were performed analogously.

### Immunofluorescence analysis of intrahepatic monocyte subsets

After deparaffinization and rehydration, slides were boiled in citrate buffer, and blocking solution (Vector Labs, Burlingame, CA) was applied. Rabbit anti-human CD14 antibody (HPA001887; Sigma), mouse anti-human CD16 (clone 2H7; MBL), or appropriate isotype control antibodies (Santa Cruz Biotechnology, Santa Cruz, CA) were detected by secondary anti-rabbit Cy-3 and anti-mouse-FITC-antibodies (Jackson ImmunoResearch, West Grove, PA). Cell nuclei were counterstained with DAPI (Vectashield, Vector Labs). Slides were then analysed by fluorescence microscopy (Zeiss, Jena, Germany). Ten bright fields per slide were randomly chosen for quantitative analysis. The investigator was blinded to the stage of fibrosis or experimental data.

### *In vitro* stimulation of monocytes

After isolation of PBMC by density gradient, 1×10^6^ cells/ml were resolved in 2 ml RPMI (Invitrogen) containing 1% penicillin-streptomycin (PAA) and 1.5% autologous serum and allowed to adhere for 35 min in a Petri dish. Non-adherent cells were discarded. Cells were then cultured for 24 h in 2 ml RPMI (5% autologous serum, 1% penicillin-streptomycin), followed by stimulation with 1 mg/ml LPS (Sigma, Hamburg, Germany) and additional incubation for 24 h.

### Cytokine/chemokine expression of human monocyte subsets *ex vivo*

PBMC of three different healthy donors were isolated by density gradient. CD14^+^CD16^−^ and CD14^+^CD16^+^ monocytes were selectively purified by MACS methodology using ‘Monocyte-isolation-Kit-II’ and ‘CD16^+^-monocytes-isolation-kit’, respectively (Miltenyi). Lymphocytes serving as control cells were isolated from PBMC after depletion of monocytes with anti-CD14 microbeads. Purity >90% was confirmed by FACS analysis. Cells were cultured in RPMI containing 10% BSA and 1% penicillin-streptomycin (PAA) for 5 days.

### Cytokine and chemokine detection

The release of cytokines/chemokines in human serum or in culture medium supernatant was measured using FlowCytomix (Bender Medsystems, Austria, Vienna). Measurements were performed in duplicates at 50 µL sample volume. Serum concentrations of MCP-1, MIP1α and MIP1β were assessed by Cytometric Bead Assay (BD) according to the manufacturer's instructions.

### Co-culture of monocyte subsets with primary human stellate cells

Human liver tissue was obtained from patients undergoing partial liver resection for metastatic liver tumors of colorectal cancer. Experimental procedures were performed according to guidelines of the local ethics committee with patient's informed consent. Primary human HSC were isolated using EGTA/collagenase perfusion and pronase incubation as described previously [35], [36]. HSC were separated from other non-parenchymal liver cells by arabinogalactan gradient ultracentrifugation, yielding HSC that were more than 90% pure and viable. 8×10^4^ HSC were seeded on uncoated plastic dishes and cultured in DMEM supplemented with 10% FCS and 100 IU/ml penicillin-streptomycin. Growth medium was changed daily for the first 4 days in culture, then every other day thereafter [37].

Monocytes of three different healthy donors were pooled, and monocyte subsets were isolated as described above. After 7 days in pre-culture primary human HSC were co-cultured for 5 days with either CD14^+^CD16^−^, CD14^+^CD16^+^ or lymphocytes (each 8×10^5^ cells/plate). As a positive control, HSC were stimulated with 5 ng/mL recombinant human TGFβ (R&D systems). *Col1A* and *Acta2* were found to be exclusively expressed in HSC (and not in PMBC, monocytes/macrophages or lymphocytes), and *col1A*, but not *Acta2*, was induced in HSC by recombinant TGFβ. Therefore, *col1A* mRNA was normalized to *Acta2* expression, in order to be able to evaluate HSC activation in cultures with mixed cell populations. In some co-culture assays, 2 ng/ml polyclonal anti-human TGFβ-Ab (sc-146; Santa Cruz) was applied [6].

### Statistical analysis

Due to the skewed distribution of most parameters assessed in patients, data are presented as median, minimum and maximum. Differences between two groups were assessed by Mann-Whitney-*U*-test, multiple comparisons between more than two groups by Kruskal-Wallis-ANOVA and Mann-Whitney-*U*-test for post hoc analysis (SPSS, Chicago, IL). Box plot graphics illustrate comparisons between subgroups, displaying a statistical summary of median, quartiles, range and extreme values. The whiskers extend from the minimum to the maximum value excluding outside (open circle) and far out (asterisk) values which are shown as separate points. Correlations between variables were assessed by Spearman rank correlation test (SPSS).

For the *ex vivo* and *in vitro* experiments, bar graphs represent the mean and the standard error of the mean (SEM). Statistical comparisons between groups were performed using Mann-Whitney-*U*-test (GraphPad Prism). P-values <0.05 were considered statistical significant.

## Supporting Information

Figure S1 Absolute numbers of circulating monocyte subsets do not differ between liver disease patients and healthy controls: Statistical analysis reveals no significant shifts in absolute numbers of CD14+CD16- and CD14+CD16+ monocytes comparing healthy controls (n = 181) with chronic liver disease patients (n = 226) or non-cirrhotic (n = 85) with cirrhotic (n = 141) patients. No significant alterations are observed between the Child's stages of cirrhosis either (Child A, n = 48; B, n = 46; C, n = 47). Box plots are displayed, where the bold black line indicates the median per group, the box represents 50% of the values, and horizontal lines show minimum and maximum values of the calculated non-outlier values; open circles indicate outlier values.
(0.07 MB PDF)

Figure S2 Increased HLA-DR expression on CD14+CD16+ monocytes in chronic liver disease: Statistical analysis reveals an increase in HLA-DR expression (mean fluorescence intensity, MFI) on CD14+CD16+ monocytes, but not on CD14+CD16- monocytes comparing healthy controls (n = 181) with chronic liver disease patients (n = 226) or non-cirrhotic (n = 85) with cirrhotic (n = 141) patients. No significant alterations are observed between the Child's stages of cirrhosis (Child A, n = 48; B, n = 46; C, n = 47). Box plots are displayed, where the bold black line indicates the median per group, the box represents 50% of the values, and horizontal lines show minimum and maximum values of the calculated non-outlier values; open circles indicate outlier values. Significant differences (U-test) are marked by *p<0.05.
(0.07 MB PDF)
