# Supplementary figures and images for: Functional Contribution of Elevated Circulating and Hepatic Non-Classical CD14+CD16+ Monocytes to Inflammation and Human Liver Fibrosis
Source: PLoS One. 2010 Jun 10;5(6):e11049. doi: 10.1371/journal.pone.0011049 (PMC2883575; doi:10.1371/journal.pone.0011049)

**Suppl. Fig. 1: Absolute numbers of circulating monocyte subsets**

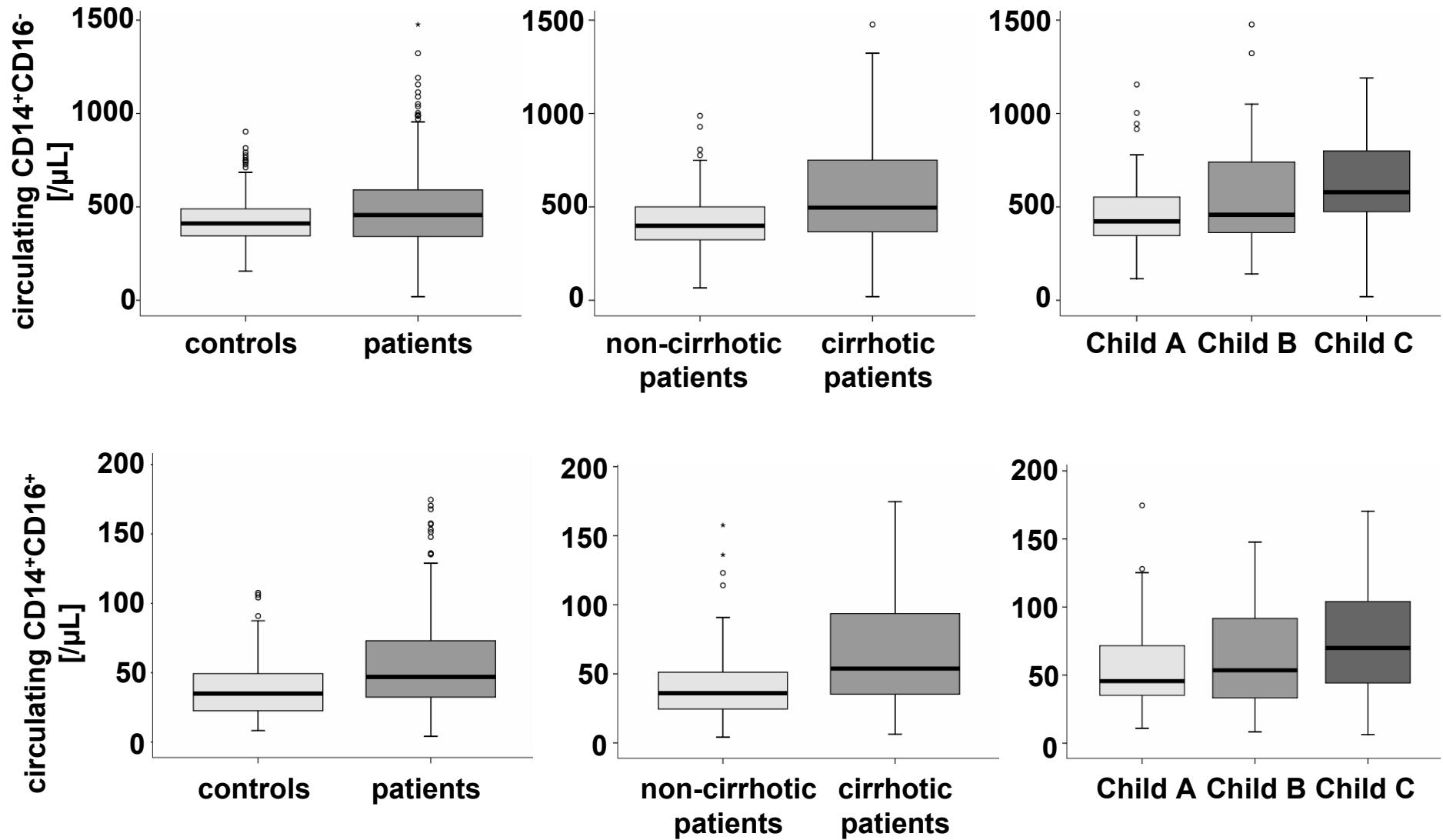

Supplement: Figure S1 — Absolute numbers of circulating monocyte subsets do not differ between liver disease patients and healthy controls: Statistical analysis reveals no significant shifts in absolute numbers of CD14+CD16- and CD14+CD16+ monocytes comparing healthy controls (n = 181) with chronic liver disease patients (n = 226) or non-cirrhotic (n = 85) with cirrhotic (n = 141) patients. No significant alterations are observed between the Child's stages of cirrhosis either (Child A, n = 48; B, n = 46; C, n = 47). Box plots are displayed, where the bold black line indicates the median per group, the box represents 50% of the values, and horizontal lines show minimum and maximum values of the calculated non-outlier values; open circles indicate outlier values. (0.07 MB PDF) [file pone.0011049.s001.pdf]
