# Supplementary material for: Functional Contribution of Elevated Circulating and Hepatic Non-Classical CD14+CD16+ Monocytes to Inflammation and Human Liver Fibrosis
Source: PLoS One. 2010 Jun 10;5(6):e11049. doi: 10.1371/journal.pone.0011049 (PMC2883575; doi:10.1371/journal.pone.0011049)
Supplement: Figure S2 — Increased HLA-DR expression on CD14+CD16+ monocytes in chronic liver disease: Statistical analysis reveals an increase in HLA-DR expression (mean fluorescence intensity, MFI) on CD14+CD16+ monocytes, but not on CD14+CD16- monocytes comparing healthy controls (n = 181) with chronic liver disease patients (n = 226) or non-cirrhotic (n = 85) with cirrhotic (n = 141) patients. No significant alterations are observed between the Child's stages of cirrhosis (Child A, n = 48; B, n = 46; C, n = 47). Box plots are displayed, where the bold black line indicates the median per group, the box represents 50% of the values, and horizontal lines show minimum and maximum values of the calculated non-outlier values; open circles indicate outlier values. Significant differences (U-test) are marked by *p<0.05. (0.07 MB PDF) [file pone.0011049.s002.pdf]

Suppl. Fig. 2: HLA-DR expression on circulating monocyte subsets

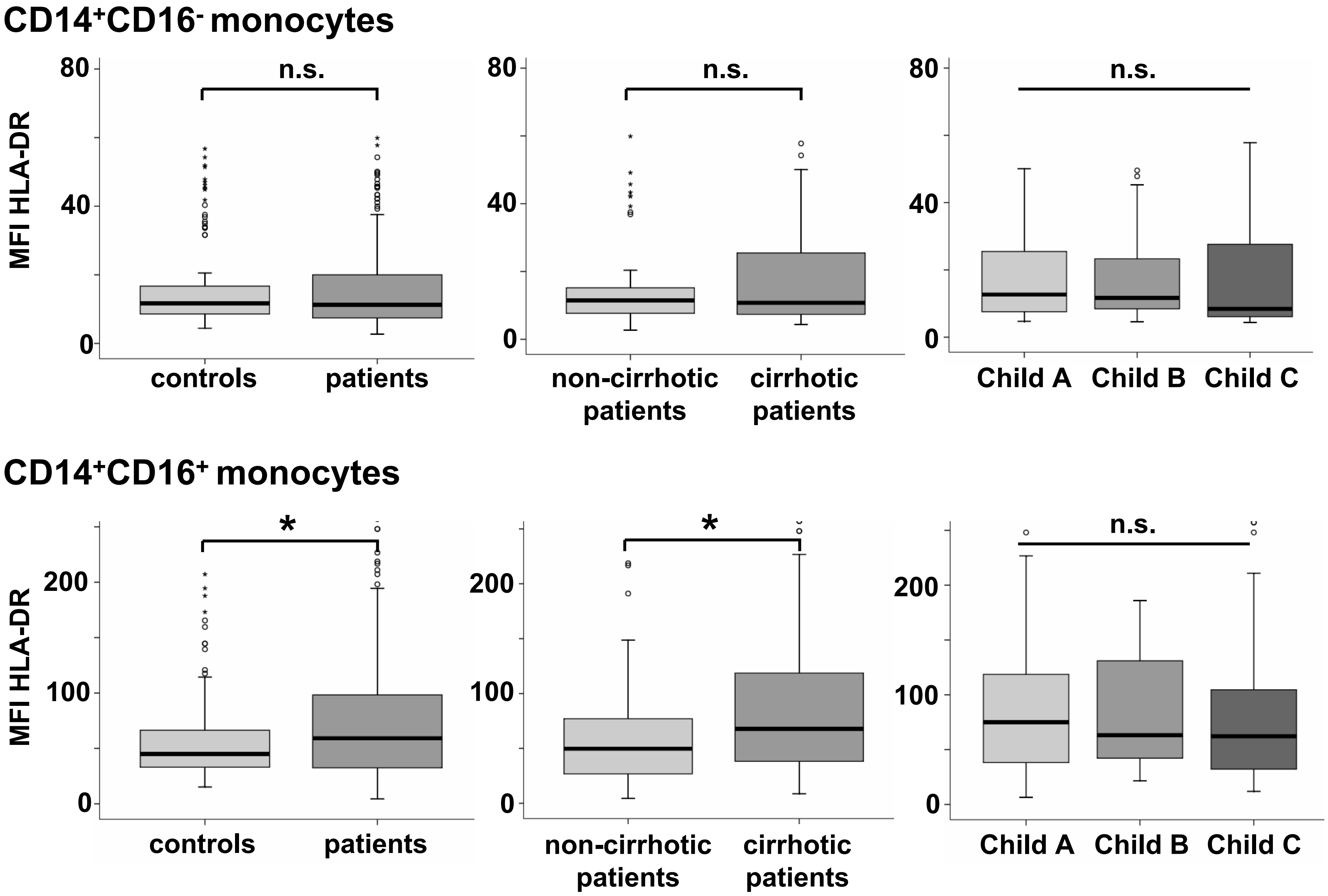

Zimmermann *et al*, Suppl. Fig. 2
